# Ecological mechanisms of microbial assembly in clonal plant *Glechoma longituba*: from soil to endosphere

**DOI:** 10.1128/aem.00336-25

**Published:** 2025-05-12

**Authors:** Yunshi Li, Na Qu, Shuang Li, Huaizhe Zhou, Ming Yue

**Affiliations:** 1 Key Laboratory of Resource Biology and Biotechnology in Western China 12657, Xi'an, China; 2 Department of Life Science, Northwest University 12657, Xi'an, China; 3 Test Center, National University of Defense Technology 58294, Xi'an, China; 4 Xi'an Botanical Garden of Shaanxi Province, Institute of Botany of Shaanxi Province 625885, Xi'an, China; University of Delaware, Lewes, Delaware, USA

**Keywords:** clonal plant, host selection, community assembly, soil-episphere-endosphere continuum

## Abstract

Climate change presents significant challenges to plant growth and reproduction. Clonal plants, with low genetic diversity, are particularly vulnerable due to their limited adaptive capacity. Plant-associated microbiomes can play a crucial role in enhancing clonal plant survival and adaptability, yet the mechanisms governing microbial community assembly along the soil-episphere-endosphere continuum remain unclear. In this study, we investigated microbial community assembly patterns in the clonal plant *Glechoma longituba*. Our findings demonstrate that the assembly of microbial communities is primarily driven by host-related factors rather than external environmental filtering. First, host selection reduced α-diversity and network complexity while increasing β-diversity and community stability. Second, the mechanisms of microbial assembly transitioned from stochastic dominance in bulk soil and epiphytic compartments to deterministic processes within endophytic niches. Third, the taxonomic structure exhibited significant turnover along the soil-episphere-endosphere continuum, accompanied by functional redundancy to maintain ecosystem functions. The results support the hypothesis that host selection optimizes the functional composition of microbial communities by reducing diversity and network complexity while ensuring the stability of key functional microorganisms. The study emphasizes the critical role of host-microbe interactions in sustaining the adaptive and functional advantages of clonal plants, offering insights into managing sustainable plant communities under climate change.

**IMPORTANCE:**

This study highlights the vital role of plant-associated microbiomes in helping clonal plants, which have low genetic diversity, adapt to climate change. By examining the clonal plant *Glechoma longituba*, the research reveals that the plant itself plays a key role in shaping its microbial communities, rather than external environmental factors. Host selection simplifies microbial diversity and network complexity but enhances community stability and functional efficiency. These findings suggest that clonal plants can optimize their microbiomes to maintain critical functions. This work provides valuable insights into how plants and microbes interact to improve resilience, offering potential strategies for managing plant communities in a changing climate. By understanding these mechanisms, we can better support sustainable ecosystems and agricultural practices in the face of global environmental challenges.

## INTRODUCTION

Climate change is transforming global ecosystems at an unprecedented rate (1). Rising temperatures, altered precipitation patterns, and increased frequency of extreme weather events pose significant threats to plant growth and reproduction (2, 3). These environmental pressures disrupt critical nutrient cycles, water availability, and habitat conditions, challenging plants' adaptability and resilience. Clonal plants, with their limited genetic diversity, face heightened risks of survival in rapidly changing environments (4). Against this backdrop, plant-associated microbiomes, including rhizosphere, phyllosphere, and endophytic microbes, play a crucial role in supporting plant survival and fitness (5, 6). Therefore, microorganisms can provide ecological and functional support for clonal plants, aiding in population stability and adaptation to environmental changes in specific habitats by enhancing nutrient uptake, promoting growth, and boosting resistance to diseases and stress.

The assembly pattern of plant microbiomes along the continuum from soil to epiphytic (plant surface) to endophytic (plant inside) microorganisms represents a complex hierarchical filtering process (7). Within this continuum, microbial communities in different niches, such as the rhizosphere, phyllosphere, and endosphere, exhibit close spatial and functional connections. However, most existing studies focus on microbial assembly in individual niches. They often overlook the connections between these niches (8–10). While current research has shed light on how plants select specific microbial communities from the soil microbial pool, there is still a lack of understanding of the dynamic changes of microbial communities in clonal plants along the soil-episphere-endosphere continuum (11). Given the critical role of microbiomes in supporting plant growth and adaptability, it is essential to gain a deeper understanding of the assembly and maintenance mechanisms of these communities in clonal plants.

The assembly of plant microbiomes is driven by the combined effects of host plants and environmental factors (12). Hosts actively select and maintain microbial communities beneficial to their growth and adaptability through selective pressures such as root exudates, tissue structures, and immune regulation (13). Simultaneously, environmental factors (e.g., soil type, temperature, moisture, and nutrient availability) provide the foundational microbial diversity, determining the composition of the potential microbial pool (14). Along the continuum from soil to epiphytic to endophytic microorganisms, hierarchical filtering mechanisms become progressively intensified, with microbial communities in different niches influenced by both local microhabitat conditions and cross-niche microbial migration (15). This complexity is pivotal for optimizing microbial community stability and functionality. Although most studies on plant microbiomes have focused on dominant plants in specific environments such as forests and agricultural systems, research on clonal plants remains relatively limited (16–18). Due to their low genetic diversity, clonal plants likely depend more on the combined effects of host selection to shape their microbiome assembly patterns. However, the key drivers and mechanisms underlying microbial community assembly along the soil-episphere-endosphere continuum remain unclear.

Therefore, this study focuses on the clonal plant *Glechoma longituba*, to elucidate the microbial community assembly patterns along the soil-episphere-endosphere continuum, especially how the host plant (clonal plant) and environmental factors synergistically affect the composition, functionality, and co-occurrence network characteristics of microbial communities. Furthermore, it aims to uncover the stability and functional redundancy of microbial communities within specific ecological niches of clonal plants, such as the rhizosphere, phyllosphere, and endosphere. This research aspires to deepen our understanding of the mechanisms underlying microbial community assembly and maintenance in clonal plants and provide strategies to optimize microbiomes for enhancing the environmental adaptability and ecological functionality of clonal plants. Ultimately, the findings will offer scientific insights for plant community management in the context of climate change.

## MATERIALS AND METHODS

### Study area and sampling

The study area was in the Jiwaji (33.85°N, 108.81°E), Chang'an District, Xi'an City, Shaanxi Province. This area is situated within the Niubeiliang Nature Reserve in the east of the Qinling Mountains, which has a temperate semi-humid monsoon climate. Summers are cool and humid, while winters are cold and dry, with an annual average temperature ranging from 2°C to 10°C and annual precipitation between 850 and 950 mm. The frost-free period lasts about 130 days. The vegetation type is a warm-temperate mixed forest of conifers and broadleaf trees, with a clear vertical distribution pattern along the elevation gradient (19). *Glechoma longituba* is commonly found in moist and water-rich environments at elevations of approximately 1,800–2,000 m in this area. Within this altitudinal zone, the predominant soil type is brown forest soil, a key zonal soil in the vertical pedogenic sequence of the Qinling Mountains' southern slope (20). In this study, four sampling sites were selected along an elevation gradient, with one site established at every 100 m interval, at elevations of 1,824 m, 1,922 m, 2,024 m, and 2,156 m. At each site, three wild *Glechoma longituba* plants were randomly collected, resulting in a total of 12 plant samples. For each plant, microbial samples were collected from five distinct niches: bulk soil, rhizosphere soil, phyllosphere, root endosphere, and shoot endosphere. To ensure comparability, we collected bulk soil samples from the top 0–10 cm layer, matching the sampling depth of the rhizosphere soil. Detailed sampling methods and protocols are provided in the supplementary materials.

### DNA extraction, Illumina PE300 sequencing, and DNA sequence availability

To comprehensively explore the structure and community composition of the clonal *Glechoma longituba* associated microbiota, we extracted total DNA from the bulk soil, rhizosphere, phyllosphere, root endosphere, and shoots endosphere samples. The specific sampling procedures for microbial samples from the five habitats are detailed in the supplementary materials.

Total DNA was extracted from microbial samples using the E.Z.N.A. Soil DNA Kit (Omega Bio-tek, Norcross, GA, USA), which is reliable and efficient in isolating high-quality DNA suitable for downstream applications. This kit provides detailed instructions for the DNA extraction process. The 18S ribosomal RNA (rRNA) gene was amplified by ITS1F and ITS2R primers (21). The 16S rRNA gene was firstly amplified by 799F and 1392R primers (22, 23) and then by 799F and 1193R primers (24). Specific information on PCR assay is provided in the supplementary information (Table S1).

Purified amplicons ran on the Illumina NextSeq platform. Detailed information on sequencing, bioinformatics, and data trimming are accessible in the supplementary information. All amplicon sequence variants (ASVs) sequenced in the control, residual plant DNA, and chimeric sequences and ASVs with less than 0.01% abundance across all samples were removed from the endophytic data set. Plant-derived taxa including Cyanobacteria, Mitochondria, Chromista, and Plantae were filtered. After this, a total of 1,085 bacterial and 954 fungal ASVs were obtained for further analysis.

### Statistical analysis and visualization

Statistical analyses were performed in R (versions 4.3.1 to 4.3.3) unless stated otherwise. Microbial α-diversity including both fungal and bacterial ASVs was calculated as Shannon index and phylogenetic diversity using Mothur (v1.30.1) (25). We performed Kruskal-Wallis or analysis of variance (ANOVA) tests to determine whether there were significant differences in diversity among different sites and different microhabitats. We determined β-diversity based on Bray-Curtis distance by principal coordinate analysis (PCoA), and the percentage of variation explained by the groups was assessed by permutational multivariate analysis of variance (PERMANOVA), analysis of similarities (ANOSIM), and multi-response permutation procedure (MRPP) test, using the Vegan package (26, 27). To explore the contribution of microhabitats, sites and their interaction due to the changes in microbial communities, we conducted a linear mixed-effects model analysis based on the glmm.hp package (28). Redundancy analysis (RDA) based on Bray-Curtis dissimilarity was performed using the ASV table of bulk soil and physicochemical properties across four sites based on the Vegan package (29). The independent contribution of each environmental variable to bulk soil community variation was explored using a hierarchical partitioning-based canonical analysis based on the rdaccaa.hp package (30). Taxa spatial dynamics and composition of endophytic communities were visualized at the phylum and class levels using a Sankey Diagram generated by the ggalluvial package (31). The top four taxa at the phylum and class level in different microhabitats were visualized using a donut chart generated by the ggforce package (32). The stability of the endophytic community was evaluated by calculating the average variation degree (AVD) index (33). The (1–AVD) index among microbiome groups was represented in the box plot. To assess correlations between the (1–AVD) index and the α-diversity indices, we used linear regression with the least squares method (34). The habitat “niche width” was calculated based on the method proposed by Levins (35) using the spaa package (36). To elucidate endophytic bacterial-fungal co-occurrence patterns in the clonal network, we constructed Spearman correlation-based co-occurrence networks using the psych package (37) and visualized it in Gephi (v10.1) (38). Network inference was conducted using ASVs that had a relative abundance exceeding 0.1% in all samples. Network key topological parameters, the degree, nodes weight degree, closeness centrality (CC), eigenvector centrality (EC), and betweenness centrality (BC) indices were represented using box plots (39). Tax4Fun2 and FUNGuild (v1.0) software were used to predict bacterial (40) and fungal (41) functions, respectively. The distribution of bacterial and fungal ASVs as well as bacterial KOs and fungal guilds shared across or unique to microhabitats was analyzed and visualized using the UpSetR package (v1.4.0) (42). The FEAST (Fast Expectation-Maximization for Source Tracking) package was utilized to perform microbial source tracking across five distinct microhabitats: bulk soil, rhizosphere, phyllosphere, shoots endosphere, and root endosphere (43).

## RESULTS

### Diversity and driving factors of the associated microbiomes in clonal plant *Glechoma longituba*

**Fig 1:**
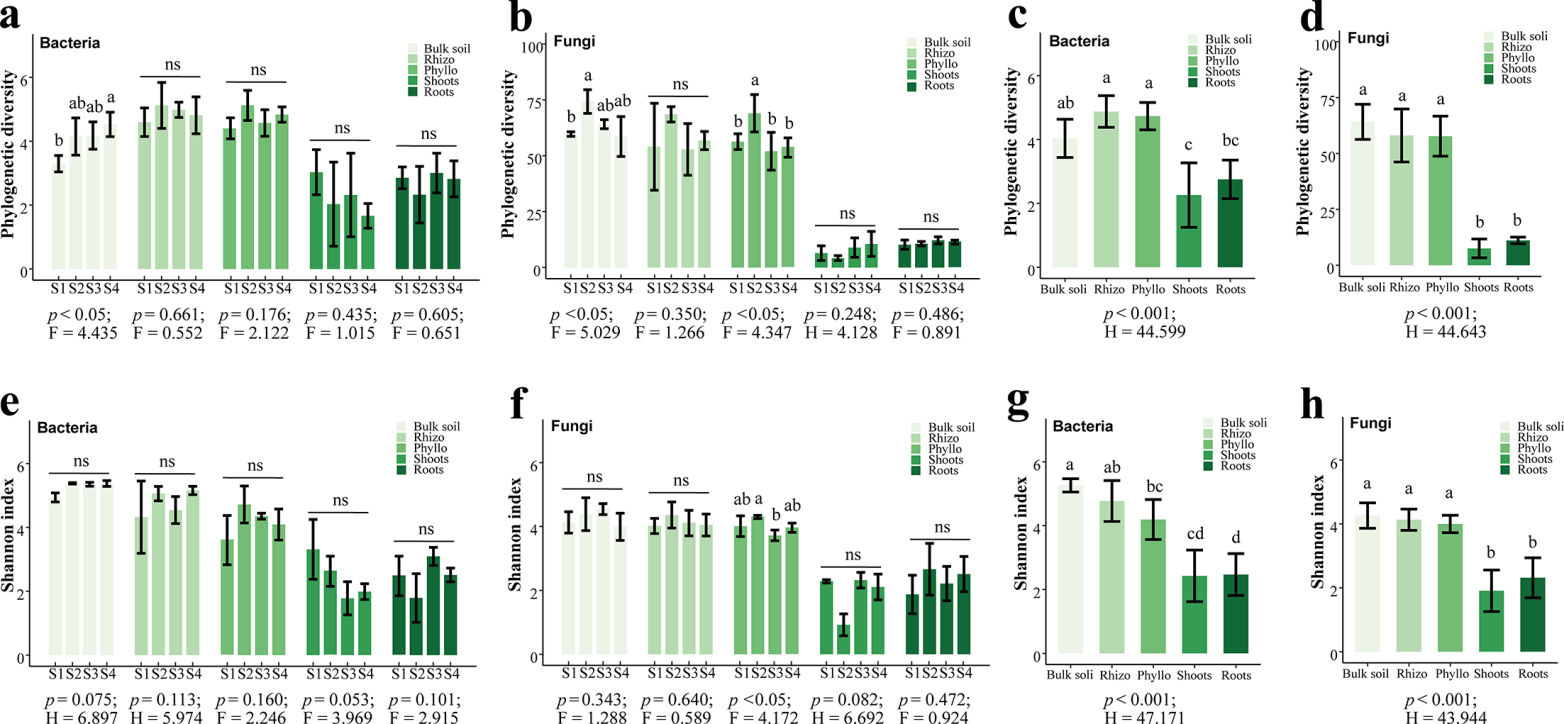
α-Diversity of the associated microbiomes in clonal plant *Glechoma longituba*. (a and **b)** Phylogenetic diversity of the microbiota of five microhabitats from four sites. (**c** and **d)** Phylogenetic diversity of the microbiota of five microhabitats aggregated from four sites.** (e** and **f)** Shannon index of the microbiota of five microhabitats from four sites. (**g** and **h)** Shannon index of the microbiota of five microhabitats aggregated from four sites. Statistical significance was determined using the Kruskal-Wallis test or ANOVA analysis. The indices (F or H and *P*-value) are displayed at the bottom of each graph. Means with the same letters are not statistically different based on the Nemenyi test or Tukey's HSD as post hoc analysis (*P* < 0.05). ns indicates no statistically significant difference between groups.

To evaluate the diversity of the associated microbiomes in clonal *Glechoma longituba*, we calculated α-diversity (Shannon index and phylogenetic diversity) at the ASV level across samples grouped into different microhabitats and sites. First, we investigated the microbiomes and physicochemical properties of bulk soil samples from the four sampling sites to elucidate the basic effects of geographic environments on the microbial community (Fig. 1; Fig. S1). Remarkably, the phylogenetic diversity revealed a significant difference in microbial composition among the four sites (Fig. 1a and b), while the Shannon indices showed no significant difference (Fig. 1e and f). Specifically, S4 and S2 had higher phylogenetic diversity than S1 and S3 in bacterial profiles and fungal profiles, respectively (*P* < 0.05). We tested eight physicochemical properties for all the bulk soil samples and observed distinct profiles, indicating that the four sites had significant differences in soil fertility (Fig. S1). Redundancy analysis (RDA) revealed that environmental factors significantly influenced soil microbial communities (*P* = 0.001) (Fig. S2). For bacterial communities (Fig. S2a), the first two components, RDA1 and RDA2, explained 38.67% and 13.04% of the variation, respectively. Similarly, for fungal communities (Fig. S2b), RDA1 and RDA2 accounted for 25.94% and 19.41% of the variation, respectively. According to hierarchical partitioning-based canonical analysis, the environmental variables explained 83.70% and 82.10% of bulk soil bacterial and fungal community variation, respectively (Fig. S3). The main contributions were found in pH (bacteria: 17.54%; fungi: 14.30%; *P* < 0.05) and TP (bacteria: 14.24%; fungi: 15.48%; *P* < 0.05) (Fig. S3).

Then, we investigated the factors influencing microbiome assembly in the clonal plant *Glechoma longituba*. Across identical habitats in different sites, the α-diversity indices of microbial communities in plant compartments, including the rhizosphere, phyllosphere, roots, and shoots, showed no significant variation (Fig. 1a, b, e and f). Notable exceptions were identified in the bacterial community, where the phylogenetic diversity (PD) index of bulk soil communities varied among sites, and in the fungal community, where both the PD and Shannon indices of bulk soil and the phyllosphere exhibited site-dependent differences (Fig. 1a, b and f). Conversely, within the same site but across distinct microhabitats, the α-diversity indices of microbial communities in all plant compartments (rhizosphere, phyllosphere, roots, and stems) were markedly reduced (Fig. S4a through d). When grouped by microhabitats as the entire collection in both the bacterial (Fig. 1c and g) and fungal assemblages (Fig. 1d and h), a significant decline in microbial α-diversity across plant compartments, encompassing the rhizosphere, phyllosphere, roots, and shoots, displayed.

**Fig 2:**
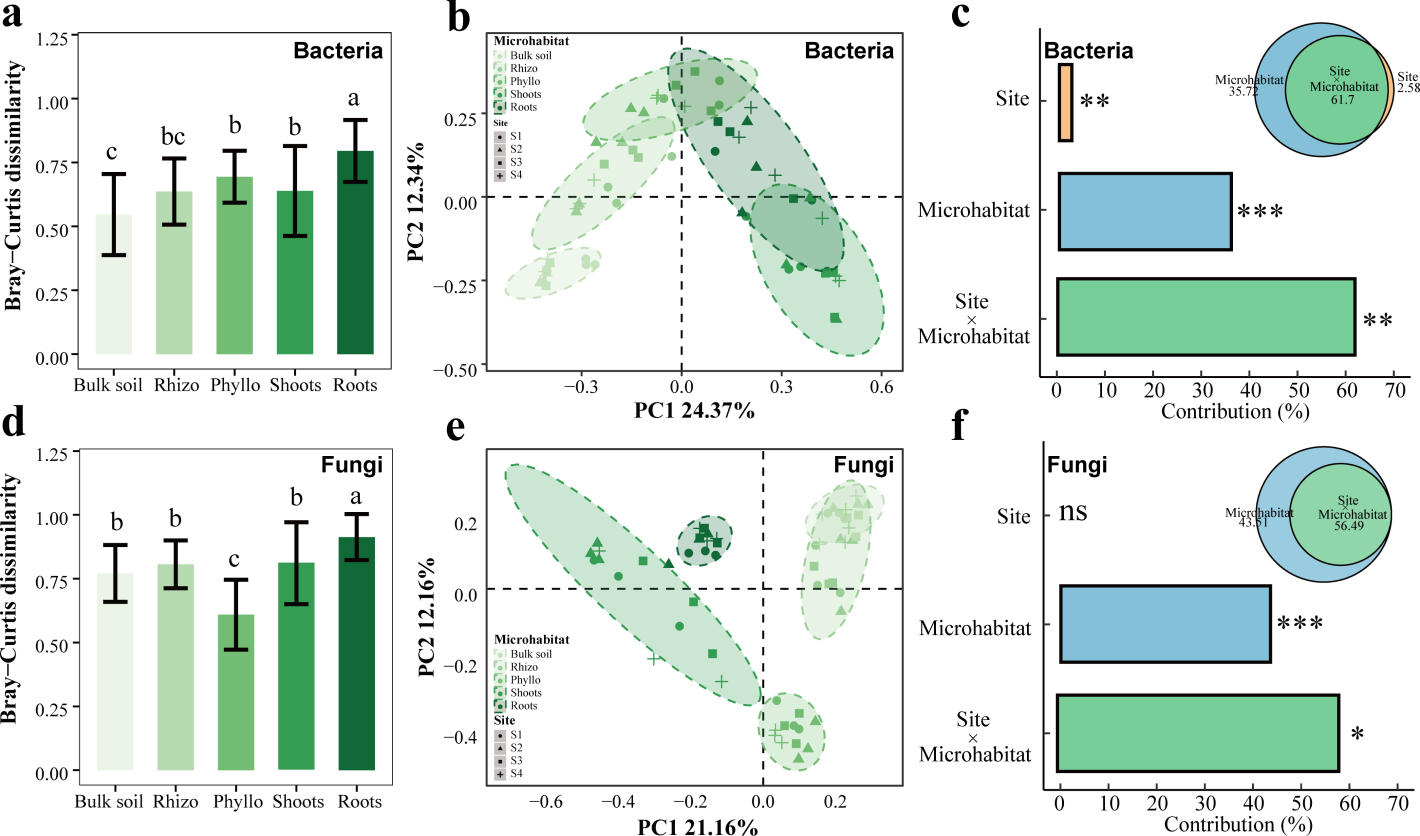
β-Diversity patterns of the associated microbiomes of clonal plant *Glechoma longituba*. (a) Bray-Curtis dissimilarity of the bacterial microbiota of five microhabitats aggregated from four sites. Means with the same letters are not statistically different based on the Nemenyi test or Tukey's HSD (*P* < 0.05). (**b)** Principal coordinates analysis (PCoA) using Bray-Curtis distances for bacterial microbiota. (**c)** The contribution of site, microhabitat, and their interaction to the changes in bacterial assemblages. (**d)** Bray-Curtis dissimilarity of the fungal microbiota of five microhabitats aggregated from four sites. (**e)** PCoA using Bray-Curtis distances for fungal microbiota. (**f)** The contribution of site, microhabitat, and their interaction to the changes in bacterial assemblages and fungal assemblages. Asterisk's indication of statistical significance: ***P < 0.001, **P < 0.01, and *P < 0.05.

Furthermore, Bray-Curtis dissimilarity analysis revealed significant differences across microhabitats in both endophytic bacterial (Fig. 2a) and fungal communities (Fig. 2d). Principal coordinate analysis (PCoA) based on Bray-Curtis dissimilarity showed clear clustering of bacterial communities by microhabitat, though some overlap across sites (S1–S4) is apparent (Fig. 2b and e). In bacterial community composition, the first two axes explained 24.37% (PC1) and 12.34% (PC2) of the variation (Fig. 2b). Variance partitioning analysis indicates that microhabitat accounts for the majority of variation (61.7%), followed by the interaction of site and microhabitat (35.72%), while the site alone contributes minimally (2.58%) (Fig. 2c). In fungal community composition, the first two axes explain 21.16% (PC1) and 12.16% (PC2) of the variance (Fig. 2e). Variance partitioning demonstrated that microhabitat remains the primary driver of fungal community variation (56.49%), while the interaction of site and microhabitat (43.31%) is secondary (Fig. 2f). Three nonparametric tests, including PERMANOVA, ANOSIM, and MRPP analyses based on Bray-Curtis and Jaccard distances, further confirmed these patterns (Table S2; Table S3).

### Taxonomic composition and dynamics from soil to endosphere

**Fig 3:**
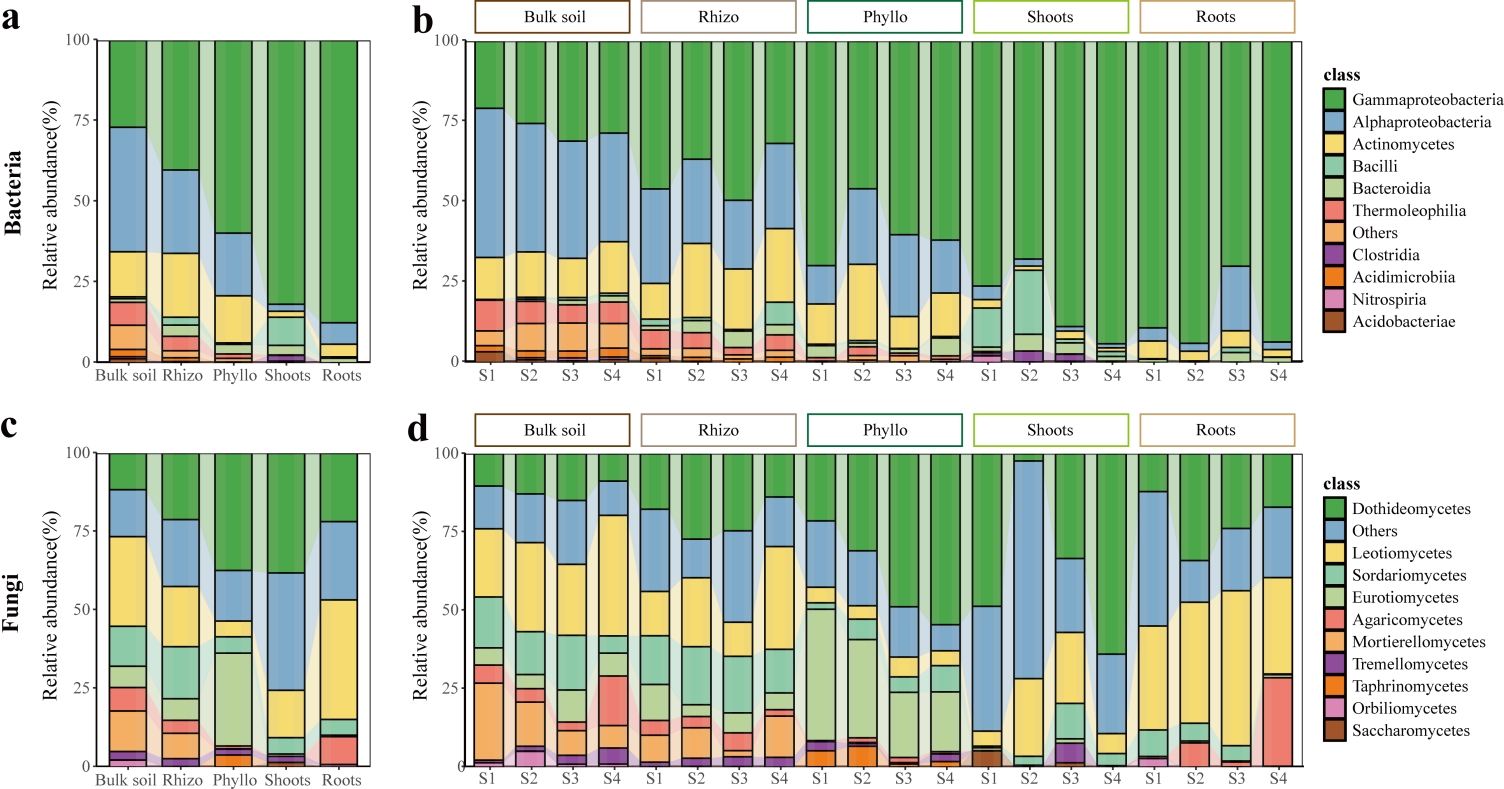
Taxonomic composition of the associated microbiomes of clonal plant *Glechoma longituba* in each microhabitat. (a and **b)** Bacterial community composition at the class level. (**c** and **d)** Fungal community composition at the class level. Compartments include bulk soil, rhizosphere (Rhizo), phyllosphere (Phyllo), shoot endosphere (Shoots), and root endosphere (Roots). Sampling sites include four: S1–S4.

After filtering, 1,538,891 high-quality reads were clustered into 1,085 bacterial ASVs and 3,272,196 high-quality reads were clustered into 954 fungal ASVs, based on the DADA2 clustering method. To characterize the microbial dynamics from soil to episphere to endosphere, we investigated microbial community composition in the five niches associated with the clonal plant *Glechoma longituba*. The bulk soil in four sampling sites served as the local microbial species pool, with the community composition of the microbial species pool illustrated in Fig. 3 and Fig. S5 and S6. In the bacterial profiles, at the class level, the bulk soil in the four sites was primarily dominated by Alphaproteobacteria (38.57%), Gammaproteobacteria (26.90%), and Actinomycetes (13.98%) (Fig. 3; Fig. S6; Table S4). At the phylum level, the bulk soil in the four sites was primarily dominated by Pseudomonadota (65.48%) and Actinomycetota (25.71%) (Fig. S5 and S6; Table S5). In the fungal profiles, at the class level, the bulk soil in the four sites was primarily dominated by Leotiomycetes (28.56%), Mortierellomycetes (12.95%), and Sordariomycetes (12.75%) (Fig. 3; Fig. S6; Table S6). At the phylum level, the bulk soil in the four sites was primarily dominated by Ascomycota (66.33%), Mortierellomycota (12.95%), and Basidiobolomycota (11.86%) (Fig. S5 and S6; Table S7).

Among the five microhabitats, the bacterial (Fig. 3a; Fig. S4a) and fungal (Fig. 3b Fig. S4b) taxonomic distributions were significantly different. Specifically, Pseudomonadota and Bacillota became significantly more dominant, whereas other phyla such as Actinomycetota became less dominant from bulk soil to plant endosphere (*P* < 0.05; Fig. S5; Table S5). Consequently, Gammaproteobacteria from Pseudomonadota and Clostridia from Bacillota were enriched progressively from bulk soil to plant endosphere, whereas classes including Alphaproteobacteria, Actinomycetes, Thermoleophilia, Acidimicrobiia, and Acidobacteriae were depleted (*P* < 0.05; Fig. 3; Table S4). For fungal communities, the two representative dominant phyla showed minimal variation across different habitats. Ascomycota was significantly more abundant in the phyllosphere compared to the other four habitats, whereas Basidiomycota exhibited significantly lower abundance in the endosphere than in the other four habitats. Along the soil-episphere-endosphere continuum, the relative abundance of Mortierellomycota and Rozellomycota gradually decreased (*P* < 0.05; Fig. S5; Table S7). Consequently, classes of Sordariomycetes, Eurotiomycetes, and Mortierellomycetes were depleted (*P* < 0.05; Fig. S5; Table S6).

### Bacterial-fungal co-occurrence networks and community stability

**Fig 4:**
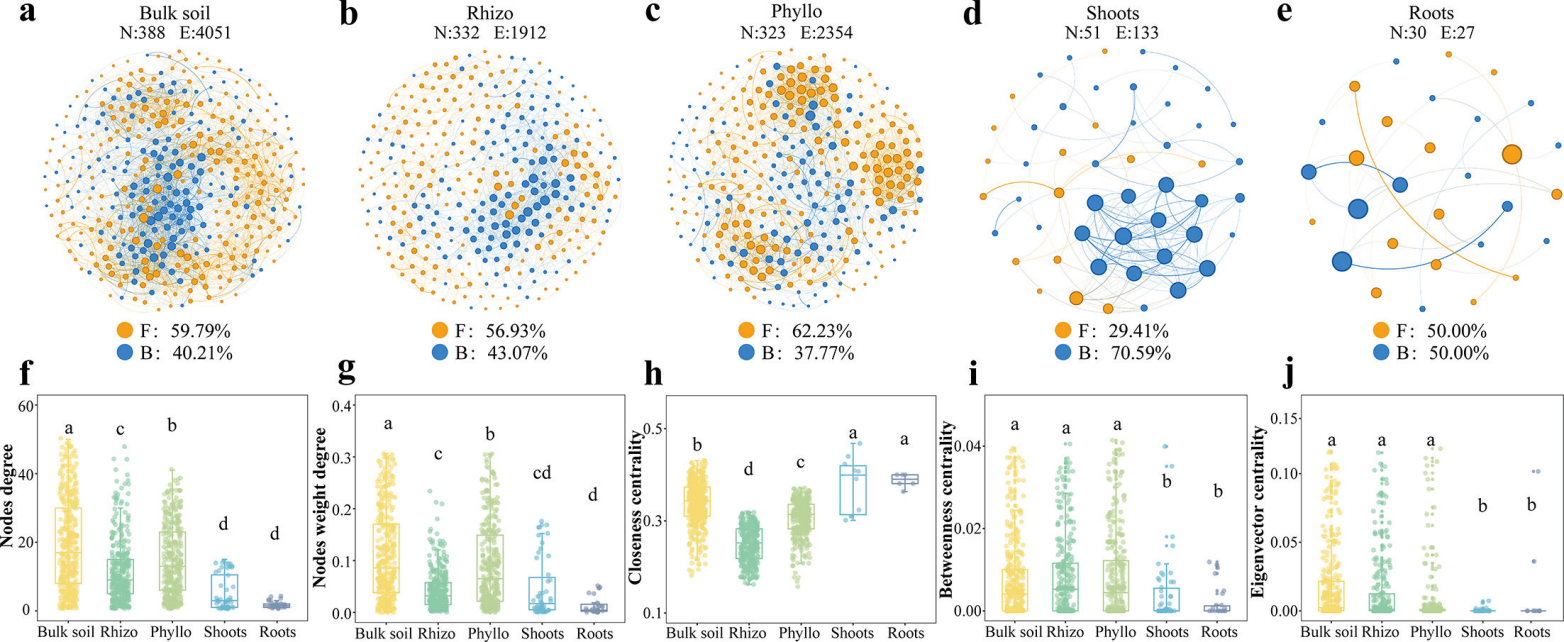
Dynamics of microbial networks. (a through e) Bacterial-fungal co-occurrence networks in each microhabitat. Each node represents a single ASV. (**f through j)** Comparison of key bacterial-fungal co-occurrence network topological properties. Means with the same letters are not statistically different based on the Nemenyi test or Tukey's HSD (*P* < 0.05).

**Fig 5:**
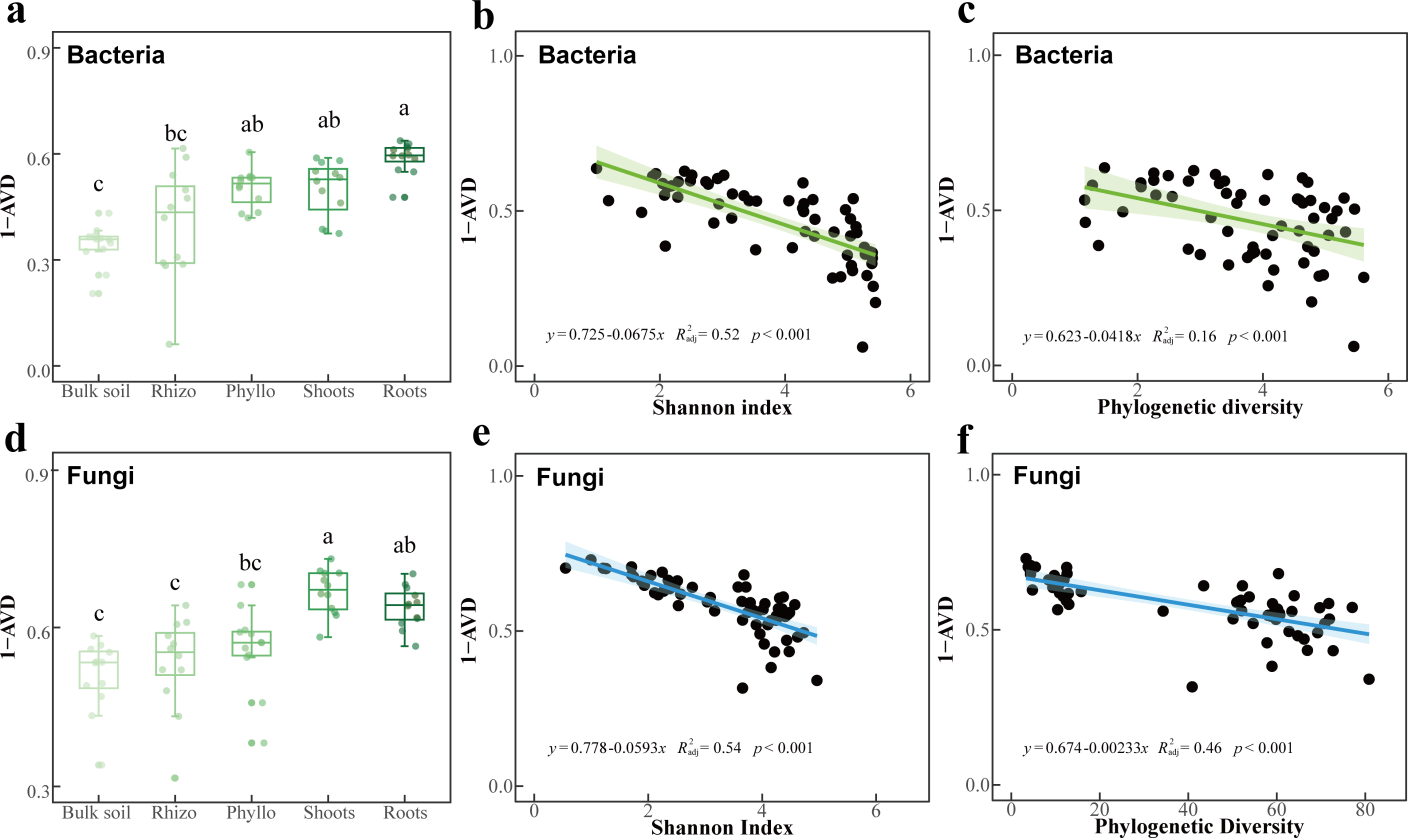
Stability of bacterial and fungal communities (1−AVD index) across plant-associated habitats and its correlation with diversity indices. (a and **d)** Stability of bacterial and fungal communities measured by average variation degree (AVD) index, shown as (1–AVD) in box plots. (**b** and **c)** Relationships between Shannon index and phylogenetic diversity with AVD index in the bacterial community. (**e** and **f)** Relationships between Shannon index and phylogenetic diversity with AVD index in fungal community. Means with the same letters are not statistically different based on the Nemenyi test or Tukey's HSD (*P* < 0.05).

To assess the microbial interconnections and community stability variations in the different microhabitats, we assessed bacterial-fungal co-occurrence networks and AVD index (Fig. 4 and 5). First, bacterial-fungal co-occurrence network complexity gradually decreased from bulk soil (with an average degree of 20.881) to shoots endosphere (1.8) (Table S8). Specifically, bacterial-fungal co-occurrence patterns of microbial communities in bulk soil, rhizosphere, and phyllosphere had higher values for node numbers, links, diameter, and average path length but had lower values for links and density (Table S8). The density in roots was the highest among all the microhabitats, and the modularity in shoots was the highest among all the microhabitats (Table S8).

The stability of bacterial and fungal communities, as measured by the 1−AVD index, varied significantly across the microhabitats (Fig. 5a and d). For bacterial communities, the 1−AVD index was lowest in bulk soil and significantly higher in phyllosphere and roots endosphere (Fig. 5a; *P* < 0.05). Similarly, fungal communities exhibited the lowest stability in bulk soil, while shoots and roots endosphere displayed the highest 1−AVD values (Fig. 5a; *P* < 0.05).

Linear regression analysis revealed significant negative correlations between the 1−AVD index and the Shannon diversity index for both bacterial and fungal communities. For bacterial communities (Fig. 5b), the regression analysis showed a strong negative relationship (R² =0.52; *P* < 0.001), indicating that higher α-diversity is associated with reduced community stability. Similarly, fungal communities (Fig. 5e) exhibited a strong negative correlation between the 1−AVD index and the Shannon index (R² =0.54; *P* < 0.001). The 1−AVD index was also negatively correlated with phylogenetic diversity for both bacterial and fungal communities, though the strength of the correlations differed. For bacterial communities, a weaker negative relationship was observed (Fig. 5c; R² =0.16; *P* < 0.001). In contrast, fungal communities showed a stronger negative correlation (Fig. 5f; R² =0.46; *P* < 0.001).

### Possible ecological assembly processes in different microhabitats

**Fig 6:**
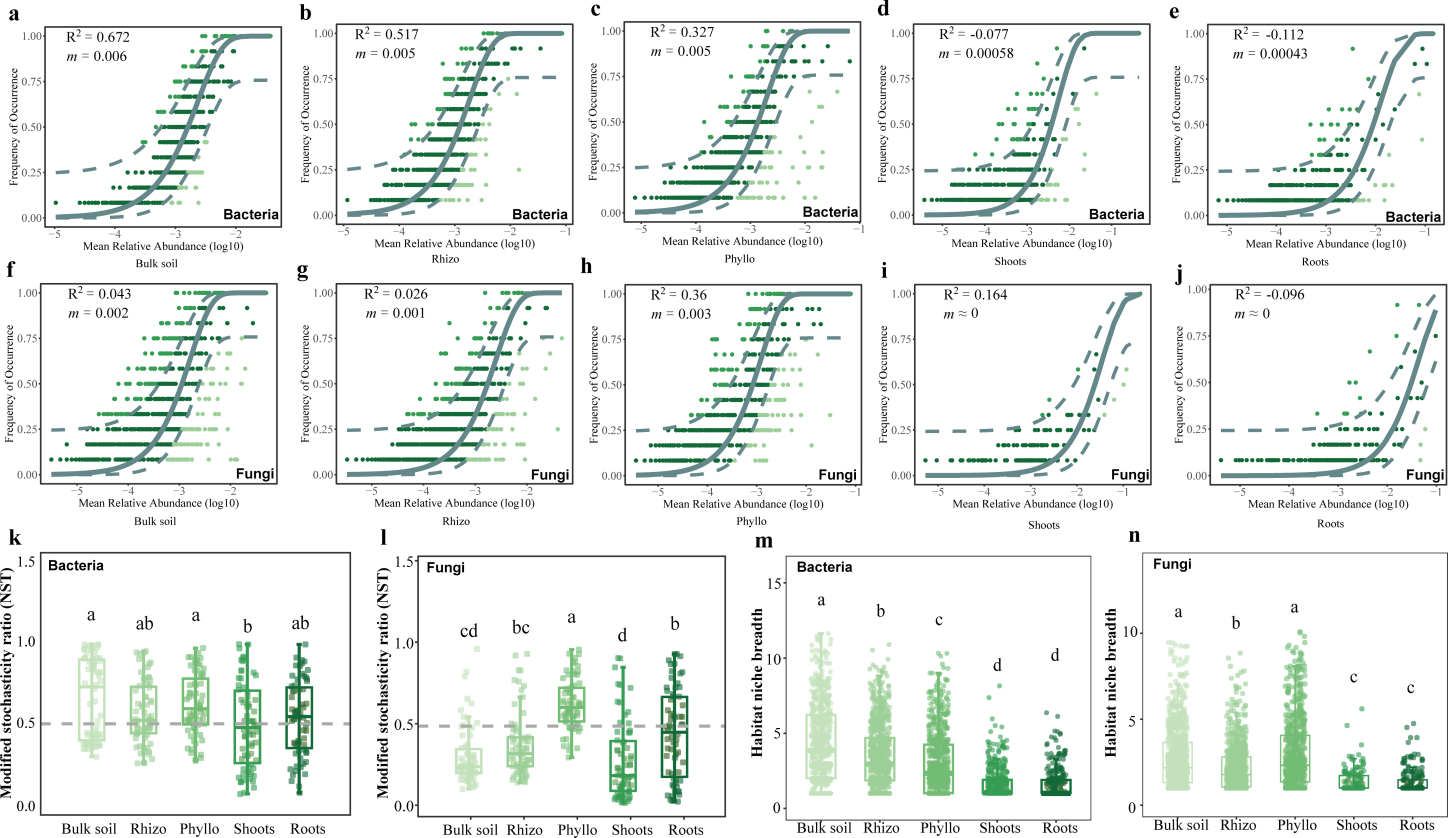
Ecological processes shaping the associated microbiomes of clonal plant *Glechoma longituba* in different microhabitats. (a through e) Neutral community model (NCM) for the stochastic assembly processes of the bacterial community in each microhabitat. (**f through j)** Neutral community model (NCM) for the stochastic assembly processes of the bacterial community in each microhabitat. R^2^ represents the fit to the neutral model, and *m* indicates the immigration rate of the metacommunity. The gray line indicates the NCM prediction with 95% confidence intervals represented in dashed lines. The points falling above and below the 95% CI are colored green and light green, respectively, and those within the interval are dark green. (**k** and **l) **The relative importance of deterministic and stochastic processes quantified by modified stochasticity ratio (NST). (**m** and **n)**Comparisons of mean Levins habitat niche breadth for bacterial and fungal communities in different microhabitats. Means with the same letters are not statistically different based on the Nemenyi test or Tukey's HSD (*P* < 0.05).

The assembly mechanisms of microbial communities across the gradient from soil to epiphytic and endophytic niches were evaluated using a neutral model (Fig. a through j). In the bulk soil, bacterial communities exhibited a strong fit to the neutral model (R^2^ = 0.672), indicating that stochastic processes primarily govern their assembly, while fungal communities showed a much weaker fit (R^2^ = 0.043), suggesting a greater influence of deterministic factors (Fig. 6a and f). In the rhizosphere, bacterial communities maintained a moderate fit (R^2^ = 0.517), whereas fungal communities showed a minimal fit (R^2^ = 0.026), reflecting a transition toward deterministic processes (Fig. 6b and g). In the phyllosphere, bacteria displayed a lower fit (R^2^ = 0.327), while fungi showed a relatively higher fit (R^2^ = 0.36), highlighting the dominance of stochastic processes in fungal assembly (Fig. 6c and h). For endophytic niches, including shoots and roots, both bacterial and fungal communities exhibited negligible fits to the neutral model (R^2^ < 0 for bacteria and R^2^ ~ 0 for fungi), indicating that deterministic factors, such as host-specific selection, strongly influence microbial assembly in these compartments (Fig. 6d and e, i and j).

The modified stochasticity ratio (NST) (Fig. 6k and l) and habitat niche breadth (Fig. 6m and n) analyses further supported these findings. NST values revealed that stochastic processes played a more significant role in epiphytic microbial communities, particularly in the phyllosphere, compared to endophytic niches (Fig. 6k and l). Habitat niche breadth analysis showed that bacterial and fungal communities in bulk soil and rhizosphere had the widest ecological niches, reflecting high flexibility, while those in shoots and roots endosphere exhibited the narrowest niches, indicating strong ecological constraints (Fig. 6m and n).

### Potential sources and overlap of microbial communities across plant compartments

**Fig 7:**
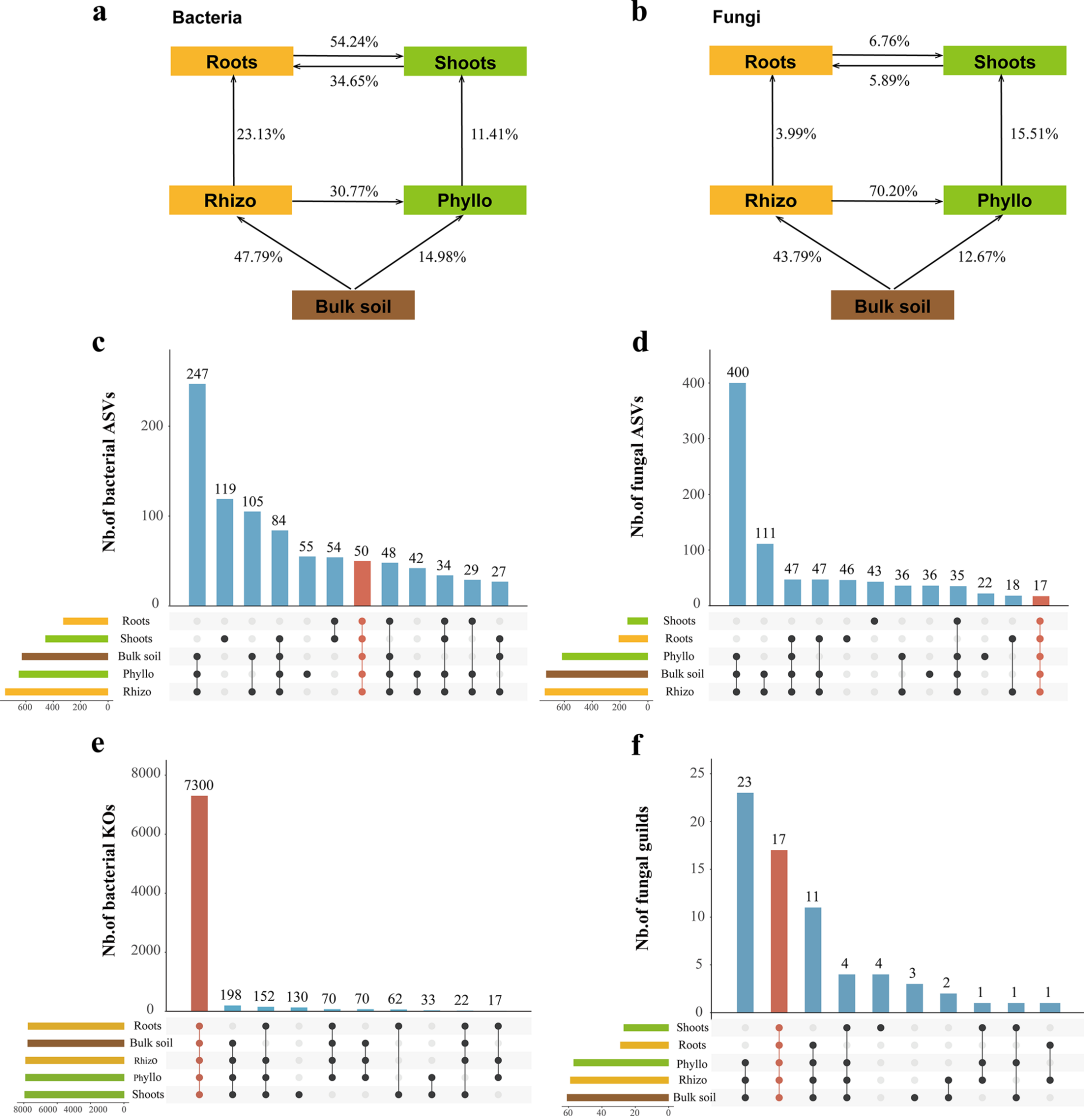
Source contributions and shared taxonomical taxa and functional groups across different compartments. (a and **b)** Source contributions of bacterial and fungal communities across compartments, with percentages indicating microbial transfers among bulk soil, rhizosphere, phyllosphere, roots endosphere, and shoots endosphere.** (c** and **d)** The upset diagram shows the total ASVs for each group, the number of unique in each group, and the number of ASVs shared between groups. (**e** and **f)** The upset diagram shows the total bacterial KOs and fungal guilds for each group, the number of KOs and guilds unique in each group, and the number of KOs and guilds shared between groups. The bar heights indicate the number of bacterial taxa shared among compartments, and the dots below represent specific combinations of compartments involved in the intersection.

To investigate the potential sources of plant endophytic microbes, we conducted a microbial tracing analysis (Fig. 7a and b). The microbial community propagates from the exterior to the interior (i.e., from the rhizosphere to the roots and the phyllosphere to the shoots) and from the lower parts to the upper parts (i.e., from the rhizosphere to the phyllosphere and from the roots to the shoots). The bulk soil acts as the microbial species reservoir, contributing 62.77% and 56.46% to the plant-associated microbiome (Fig. 7a and b). During the propagation process, each compartment selectively filters these microbes, ultimately converging in the shoot endosphere. In the bacterial community, 65.65% of the endophytic bacteria in the shoots originated from the phyllosphere and root endosphere, while 57.78% of the endophytic bacteria in the roots originated from the rhizosphere and shoot endosphere (Fig. 7a). In the fungal community, 22.27% of the endophytic fungi in the shoots endosphere originated from the phyllosphere and root endosphere, while 9.88% of the endophytic fungi in the root endosphere originated from the rhizosphere and shoot endosphere (Fig. 7b).

To evaluate the distribution of ASVs (Fig. 7c and d) and functional groups (Fig. 7e and f) across different compartments (bulk soil, rhizosphere, phyllosphere, roots, and shoots), we constructed upset diagrams. In bacterial ASV profiles, the analysis showed that 7.9% (Bulk soil), 6.7% (Rhizo), 7.7% (Phyllo), 10.9% (Shoots), and 15.3% (Roots) of the ASVs were common (area in red) to the five microhabitats (Fig. 7c). In fungal ASV profiles, the analysis showed that 2.3% (Bulk soil), 2.3% (Rhizo), 2.8% (Phyllo), 11.6% (Shoots), and 8.1% (Roots) ASVs were common (area in red) to the five microhabitats (Fig. 7d). In total, the bacterial profiles contain 50 shared ASVs, while the fungal profiles have 17 shared ASVs. However, the proportion of the overlapped functional groups was much higher than those shared by ASVs. In 16S data sets, the overlapped bacterial KOs were up to 94.83% (Bulk soil), 92.77% (Rhizo), 92.70% (Phyllo), 91.90% (Shoots), and 95.14% (Roots), respectively (Fig. 7e). In ITS data sets, the overlapped functional guilds were up to 27.9% (Bulk soil), 28.8% (Rhizo), 29.8% (Phyllo), 63.0% (Shoots), and 58.6% (Roots), respectively (Fig. 7f).

## DISCUSSION

### Host-driven microbial community assembly in *Glechoma longituba* outweighs environmental influence

Our results indicate that microbial community assembly in the clonal plant *Glechoma longituba* is predominantly influenced by plant-associated niches rather than by external environmental factors. First, microhabitat type significantly shapes the α- and β-diversities of microbiomes associated with *Glechoma longituba* (Fig. 1 and 2). Distinct plant compartments, such as the rhizosphere, phyllosphere, and endosphere, provide unique physicochemical conditions (e.g., pH and nutrient availability) that drive microbial differentiation (44, 45). The stability of these microhabitats primarily influences α-diversity, consistent with previous studies (46, 47). Notably, internal heterogeneity between microhabitats (e.g., rhizosphere vs phyllosphere) has a greater impact on microbial composition than geographic variation. Across diverse locations, the β-diversity between distinct microhabitats remains higher than within-site variation (Fig. 1; Fig. S3) (48). This pattern results from plant-driven microbial selection, including root exudation and immune responses, which homogenize microbiome composition across different geographic regions (49).

Second, microbial assembly shifts from stochastic-dominated processes in bulk soil and epiphytic niches to deterministic-dominated processes in endophytic niches, governed primarily by host selection (Fig. 6). The high environmental heterogeneity in bulk soil and epiphytic habitats (44) favors stochastic colonization and ecological drift (50–52) with minimal host filtering and high dispersal rates amplifying stochastic assembly (53). In contrast, endophytic niches (e.g., internal root, stem, and leaf tissues) are strongly influenced by host-mediated immune responses and chemical signaling (54). Microorganisms colonizing these niches must adapt to specific physicochemical constraints, such as low oxygen levels and specialized metabolic substrates. Host-driven selection reduces stochasticity by favoring symbiotic microbes that enhance nutrient acquisition and pathogen defense while excluding incompatible species (55–57). This host-driven selection ultimately dictates endophytic community structure (58).

Notably, clonal plants can share water, nutrients, and signaling molecules among ramets, such as clover (*Trifolium repens*), and poplar trees (*Populus tremula*), which may lead to a more homogenized microbial composition across different microhabitats (44, 59). Additionally, clonal integration allows clonal plants to buffer localized environmental stress, potentially reducing the effects of stochastic drift in microbial communities while enhancing host selection, thereby making microbial assembly more deterministic (60). Therefore, our findings can theoretically be extended to other clonal plant species, particularly those that rely on clonal propagation and exhibit long-term genetic stability, as their associations with symbiotic microorganisms may be more persistent and structurally stable (61). In contrast, non-clonal plants, lacking clonal integration and a stable genetic background, undergo more frequent generational turnover, making their microbiome assembly processes more susceptible to external environmental influences and genetic variation.

### Host selection reduced α-diversity and network complexity while enhancing β-diversity and community stability

Our results demonstrate that as microorganisms transition from bulk soil to epiphytic and endophytic niches, α-diversity and network complexity decline, whereas β-diversity and community stability increase (Fig. 1, 2, 4, and 6). This shift reflects a transition from a diverse, resource-rich environment to a selective, resource-limited habitat shaped by host filtering. Host plants selectively influence microbial communities through root exudates (62), signaling molecules (63), and immune responses (64), retaining only beneficial microbes. This filtering process reduces α-diversity within niches by limiting species recruitment while enhancing β-diversity through increased differentiation between microhabitats (22, 65). As microbes transition inward, host selection intensifies, excluding non-functional or weakly interacting taxa (66).

The decline in network complexity results from reduced species redundancy and fewer interspecies interactions, streamlining microbial communities into functionally cohesive subsets. Although decreased diversity and complexity are often viewed as disadvantages, our findings suggest that they enhance community stability, promoting functional efficiency and adaptation (Fig. 5 and 7). This aligns with the diversity-stability trade-off theory, where lower diversity increases stability by reducing competition and maximizing functional redundancy (33, 67). Additionally, simplified microbial networks buffer against external disturbances, improving resilience to environmental fluctuations (68). The enclosed endophytic niche, strongly regulated by the host, insulates microbial communities from external variability, further enhancing stability (44). For clonal plants, increased microbial stability supports adaptation to stressors such as drought, nutrient scarcity, and pathogen invasion, providing a key survival strategy. Thus, while host selection reduces microbial diversity and network complexity, it enhances community stability, optimizing host-microbe interactions in resource-limited and stressful environments.

### Taxonomic turnover, functional redundancy, and conserved core microbiota along the soil-to-endosphere continuum

Across the soil-episphere-endosphere continuum, microbial taxonomic composition exhibits significant turnover across habitats (Fig. 3; Fig. S5 and S6). Bulk soil is dominated by microbial groups such as Pseudomonadota and Actinomycetoma, which thrive under nutrient-rich and oxygen-variable conditions. In contrast, Bacillota and Alphaproteobacteria dominate rhizosphere communities as r-strategists capable of utilizing diverse root-derived carbon substrates (69–72). Gammaproteobacteria are prevalent in the root and shoot endosphere, playing critical roles in host growth regulation and pathogen suppression (73, 74). This taxonomic turnover is primarily driven by environmental selection pressures, including nutrient availability, oxygen gradients, and host specificity, which promote niche differentiation (13, 75). These shifts highlight the ecological flexibility of microbes in adapting to environmental constraints.

Despite distinct taxonomic compositions across microhabitats, functional redundancy ensures the continuity of key ecosystem functions (Fig. 7e and f). Our results confirm that, along the soil-episphere-endosphere continuum, essential microbial functions remain conserved, even as taxonomic composition shifts. Functionally similar microbes compensate for taxonomic losses, maintaining processes such as nutrient cycling and energy flow (5, 76). Functional redundancy is primarily shaped by plant selection and habitat conditions rather than random ecological drift (77, 78). This suggests that plants indirectly shape microbial communities with similar functional attributes by modifying habitat conditions rather than selecting specific taxa.

The conserved core microbial taxa along the soil-to-endosphere continuum further underscores the host plant's ability to recruit and sustain specific microorganisms across diverse niches (6, 78, 79). The soil microbiome serves as a foundational diversity pool, while host-mediated selective pressures retain key taxa essential for plant health and function (80). The core microbiota enhances plant growth, disease resistance, and adaptive evolution, acting as key ecological partners in sustaining host survival (81). Insights from culturable microorganisms provide valuable functional information for further ecological exploration and biotechnological applications.

Key bacterial genera within the conserved microbiota include nitrogen-fixing taxa such as *Allorhizobium-Neorhizobium-Pararhizobium-Rhizobium* (82), *Bradyrhizobium* (83), and *Pelomonas* (84, 85). *Bradyrhizobium* and *Pseudorhodoplanes* also contribute to sulfur metabolism (83, 86), while *Bradyrhizobium* and *Pelomonas* species produce indole-3-acetic acid (IAA) (84). Other genera, including *Aureimonas* (87), *Pseudoxanthomonas* (88), *Flavobacterium* (89), and *Kribbella* (90), exhibit antimicrobial properties. *Microbacterium* species enhance cold resistance and promote plant growth (91), while *Massilia* species enriched in soybean rhizosphere promote root growth and significantly increase soybean oil content (92). Fungal members of the core microbiota also play crucial roles. *Colletotrichum* and *Tetracladium* function as endophytic fungi, enhancing phosphorus uptake (93) and increasing crop yield (94). Fungal decomposers such as *Cadophora* (95) and *Paraphoma* (96) contribute to organic matter breakdown. Some *Dactylaria* species produce bioactive compounds that attract and kill nematodes (97), while *Cladosporium* (98) and *Didymella* generate antibacterial activity (99).

### Conclusion

We discovered that the assembly of microbial communities in the clonal plant *Glechoma longituba* is primarily driven by host-related factors, rather than being shaped by environmental filtering. First, host selection reduced α-diversity and network complexity but increased β-diversity and community stability. Second, microbial assembly mechanisms shifted from stochastic dominance in the bulk soil and epiphytic compartments to deterministic processes within the endophytic niches. Third, there was a clear turnover in taxonomic structure along the soil-episphere-endosphere continuum, with functional redundancy ensuring the maintenance of ecosystem functions. The results of our study support the hypothesis that host selection optimizes the functional composition of microbial communities by reducing diversity and network complexity while ensuring the stability of key functional microorganisms. The study results deepen our understanding of the symbiotic relationship between hosts and core microbial taxa in environmental adaptation, providing important insights into how clonal plants maintain adaptability and functional advantages in specific environments under the context of climate change.

## Data Availability

Raw data are accessible in NCBI under BioProject no. PRJNA1193280.

## AFTER EPUB

*[This article was published on 12 May 2025 with incorrect reference 28. The reference was corrected in the current version, posted on 21 May 2025.]*

## SUPPLEMENTAL MATERIAL

Supplemental material aem.00336-25-s0001.docx Supplemental methods, Fig. S1 to S6, and Tables S1 to S8.

ASM does not own the copyrights to Supplemental Material that may be linked to, or accessed through, an article. The authors have granted ASM a non-exclusive, world-wide license to publish the Supplemental Material files. Please contact the corresponding author directly for reuse.
