# Supplementary material for: Ecological mechanisms of microbial assembly in clonal plant Glechoma longituba: from soil to endosphere
Source: Appl Environ Microbiol. 2025 May 12;91(6):e00336-25. doi: 10.1128/aem.00336-25 (PMC12175508; doi:10.1128/aem.00336-25)
Supplement: Supplemental material — Supplemental methods, Fig. S1 to S6, and Tables S1 to S8. [file aem.00336-25-s0001.docx]

***Supplementary Information for***

**Ecological Mechanisms of Microbial Assembly in Clonal Plant *Glechoma longituba*: From Soil to Endosphere**

Yunshi Li^a,b^, Na Qu^a,b^, Shuang Li^a,b^, Huaizhe Zhou^c^, MingYue ^a,b,d,*^

*^a^ Key Laboratory of Resource Biology and Biotechnology in Western China, Xi’an 710069, China*

*^b^ Department of Life Science, Northwest University, Xi’an 710069, China*

*^c^ Test Center, National University of Defense Technology, Xi’an 710106, China*

*^d^ Xi’an Botanical Garden of Shaanxi Province/Institute of Botany of Shaanxi Province, Xi’an 710106, China*

* **Corresponding author:** Ming Yue

Key Laboratory of Resource Biology and Biotechnology in Western China, Xi’an 710069, China.

Tel.: +86 137 7245 7901.

E-mail address: [yueming@nwu.edu.cn](mailto:yueming@nwu.edu.cn)

**Content of this file**

Supplementary methods

Supplementary results: Fig. S1; Fig. S2; Fig. S3; Fig. S4; Fig. S5; Fig. S6; Table S1; Table S2; Table S3; Table S4; Table S5; Table S6; Table S7; Table S8.

**Supplementary methods**

**Sampling methods for plant associated microbiome**

The sampling procedure for plant surface microbial samples: First, for phyllosphere microbial samples, 10–15 g of leaves was placed in a 100 mL sterile plastic centrifuge tube containing 50 mL of sterile phosphate-buffered saline (PBS). The suspension was vortexed six times over 5 minutes, each vortex lasting 10 seconds. The leaves were then removed and transferred to sterile resealable bags for unified processing. The PBS suspension was filtered using a 100 mL sterile syringe onto a 50 mm-diameter, 2 μm-pore-size membrane filter (Millipore, Billerica, MA). The filters were placed into sterile centrifuge tubes, kept at low temperatures during transport to the laboratory, and stored at −80 °C prior to processing. For each sample, 3–5 replicates were collected as backups. Second, for rhizosphere microbial samples, if a significant amount of soil adhered to the root surface, it was gently removed using sterile cotton swabs. Subsequently, 3–5 g of roots were collected and processed following the same steps as for the phyllosphere microbial samples. The PBS solution used for sampling (0.010 mol/L) was prepared with 8 g/L sodium chloride (NaCl), 0.2 g/L potassium chloride (KCl), 1.44 g/L disodium hydrogen phosphate (Na₂HPO₄), and 0.24 g/L potassium dihydrogen phosphate (KH₂PO₄), with the pH adjusted to 7.2 using sodium hydroxide (NaOH).

Sampling methods for plant endophytic microbial samples: To isolate plant endophytic microorganisms, surface microbial cells were removed following the above-described methods. The obtained leaves or roots were then thoroughly washed with sterile water and subjected to surface sterilization. Under sterile conditions, the cleaned, healthy plant roots and leaves were cut into small pieces (1 cm × 1 cm or smaller) and placed in sterile 50 mL centrifuge tubes. The root samples were cleaned with 1% sodium hypochlorite for 1 minute, and leaf samples were treated with 1% sodium hypochlorite for 1 minute as well (Kim et al., 2020). Each sample was then rinsed five to six times with sterile water, and excess water was removed using sterilized filter paper. To ensure effective surface disinfection, 100 µl of the final rinse water was applied to an R2A agar plate as a control. The absence of colonies on the control plate confirmed thorough disinfection, ensuring the microorganisms collected were endophytes rather than airborne or epiphytic.

Sampling methods for bulk soil microbial samples: For soil microbial samples, 0.4 g of soil was measured and processed for DNA extraction using the PowerSoil DNA Extraction Kit, following the manufacturer’s instructions.

**Soil physicochemical characteristics**

The physical and chemical properties of the analyzed samples are summarized in Fig. S1. Kruska-Wallis or ANOVA tests were performed to determine whether there were significant differences in soil physicochemical characteristics among different sites. The total organic carbon (TOC), total nitrogen contents (TN), and total phosphorus (TP) were quantified with an automatic element analyzer (Elementar Vario EL, Germany) (Liebner et al., 2009), and total potassium (TK) was measured by flame atomic (Liu et al., 2021). The soil potential of hydrogen (pH) was measured in a 1:2.5 soil/H_2_O suspension using a waterproof pH/ORP meter (Li et al., 2018). The total dissolved solids (TDS), electric conductivity (EC) and the total soil salinity (Salinity) were measured with a conductivity/TDS meter (Atekwana et al., 2004). The soil water content (SMC) was determined gravimetrically after the soil was dried in an oven at 105 ℃ for 12 h (Liu et al., 2010).

**Sequencing, bioinformatics and data trimming**

After demultiplexing, the resulting sequences were quality filtered with fastp (version 0.19.6) (Chen et al., 2018) and merged with FLASH (version 1.2.11) (Tanja et al., 2011). Then the high-quality sequences were de-noised using DADA2 plugin in the Qiime2 (version 2020.2) pipeline with recommended parameters, which obtains single nucleotide resolution based on error profiles within samples (Callahan et al., 2016). DADA2 denoised sequences are usually called amplicon sequence variants (ASVs). Taxonomic assignment of ASVs was performed using the Naive bayes consensus taxonomy classifier implemented in Qiime2 (Wang et al., 2007). Sequences of bacterial and fungal were assigned to taxonomic based on the taxonomy database of silva138/16s _bacteria and unite8.0/its_fungi, respectively (Lu et al., 2023).

**References**

Atekwana, E. A., Atekwana, E. A., Rowe, R. S., Werkema, D. D. Jr., and Legall, F. D. (2004). The relationship of total dissolved solids measurements to bulk electrical conductivity in an aquifer contaminated with hydrocarbon. J. Appl. Geophys. 56, 281-294.

Callahan, B. J., McMurdie, P. J., Rosen, M. J., Han, A. W., Johnson, A. J. A., & Holmes, S. P. (2016). DADA2: High-resolution sample inference from Illumina amplicon data. Nature methods, 13(7), 581-583.

Chen, S., Zhou, Y., Chen, Y., & Gu, J. (2018). fastp: an ultra-fast all-in-one FASTQ preprocessor. Bioinformatics, 34(17), i884-i890.

Kim, H., Lee, K. K., Jeon, J., Harris, W. A., & Lee, Y. H. (2020). Domestication of Oryza species eco-evolutionarily shapes bacterial and fungal communities in rice seed. Microbiome, 8(1), 20.

Li, Y., Wu, X., Chen, T., Wang, W., Liu, G., Zhang, W., ... & Zhang, G. (2018). Plant phenotypic traits eventually shape its microbiota: a common garden test. Frontiers in microbiology, 9, 2479.

Liebner, S., Rublack, K., Stuehrmann, T., and Wagner, D. (2009). Diversity of aerobic methanotrophic bacteria in a permafrost active layer soil of the Lena Delta, Siberia. Microb. Ecol. 57, 25-35.

Liu, Z., Fu, B., Zheng, X., and Liu, G. (2010). Plant biomass, soil water content and soil N: P ratio regulating soil microbial functional diversity in a temperate steppe: a regional scale study. Soil Biol. Biochem. 42, 445–450.

Liu, J., Shu, A., Song, W., Shi, W., Li, M., Zhang, W., ... & Gao, Z. (2021). Long-term organic fertilizer substitution increases rice yield by improving soil properties and regulating soil bacteria. Geoderma, 404, 115287.

Lu, D. C., Wang, F. Q., Amann, R. I., Teeling, H., & Du, Z. J. (2023). Epiphytic common core bacteria in the microbiomes of co-located green (Ulva), brown (*Saccharina*) and red (*Grateloupia*, *Gelidium*) macroalgae. Microbiome, 11(1), 126.

Tanja, Mago, Steven, et al. (2011). FLASH: fast length adjustment of short reads to improve genome assemblies. Bioinformatics, 27(21):2957‐2963.

Wang, Q., Garrity, G. M., Tiedje, J. M., & Cole, J. R. (2007). Naive Bayesian classifier for rapid assignment of rRNA sequences into the new bacterial taxonomy. Applied and environmental microbiology, 73(16), 5261-5267.

**Supplementary results**

**Fig. S1.** Physicochemical properties of bulk soil samples from four sites. Means with the same letters are not statistically different based on Nemenyi or Tukey’s HSD test (*p* < 0.05).

**Fig. S2.** Redundancy analysis (RDA) based on Bray-Curtis dissimilarity was performed using the ASV table of bulk soil and physicochemical properties across four sites. **a** in bacterial profiles. **b** in fungal profiles. RDA1 and RDA2 represent the first and second components of the analysis, respectively. Point shapes and colors are used to highlight different sites. Microbial community dissimilarities among groups were tested for significance using adonis test (*p* = 0.001).

**
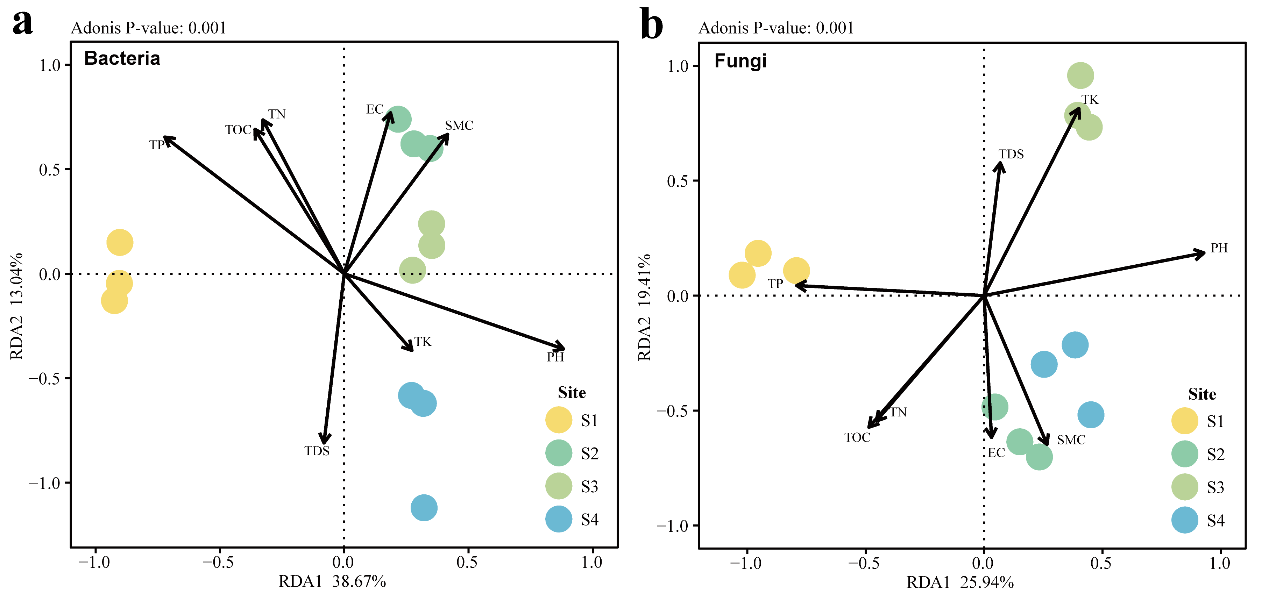
**

**Fig. S3.** Quantifying the independent contribution of each environmental variable to community variation using a hierarchical partitioning-based canonical analysis.

**Fig. S4.** Alpha diversity of the associated microbiomes in clonal plant *Glechoma longituba*. **a** and **b** Phylogenetic diversity of the microbiota of five microhabitats in different sites. **c** and **d** Shannon index of the microbiota of five microhabitats in different sites. Means with the same letters are not statistically different based on Nemenyi test or Tukey’s HSD as post hoc analysis (*p* < 0.05). ns indicates no statistically significant difference between groups.

**Fig.S5.** Taxonomic composition of the associated microbiomes of clonal plant *Glechoma longituba* in each microhabitat. **a** and **b** Bacterial community composition at phylum level. **c** and **d** Fungal community composition at phylum level. Compartments include bulk soil, rhizosphere (Rhizo), phyllosphere (Phyllo), shoot endosphere (Shoots), and root endosphere (Roots). Sampling sites include four: S1–S4.

**Fig. S6.** Donut chart of the top 4 phylum (**a**, **c**) and classes (**b**, **d**) of microbiota in each microhabitat.

**Table S1** The composition of PCR assay and thermal cycling conditions.

| **Target gene** | **Primers** | **PCR assay** | **Thermal cycling conditions** |
| --- | --- | --- | --- |
| Bacterial 16S rRNA gene | 799F: 5’-AACMGGATTAGATACCCKG-3’; 1392R: 5’-ACGGGCGGTGTGTRC-3’ | 20 μl reactions consisted of 10 μl 2×Pro Taq, 0.8μl forward primer(5 μM), 0.8μl reverse primer(5 μM), 10ng template DNA, ddH_2_O. | 3 minutes at 95°C, then 27 cycles × (30 seconds at 95°C; 30 seconds at 55 °C; 45 seconds at 72°C), finally 10 minutes at 72°C, 10 °C until halted by user |
|  | 799F: 5’-AACMGGATTAGATACCCKG-3’; 1193R: 5’-ACGTCATCCCCACCTTCC-3’ | 20 μl reactions consisted of 10 μl 2×Pro Taq, 0.8μl forward primer(5 μM), 0.8μl reverse primer(5 μM), 10ng template DNA, ddH_2_O. | 3 minutes at 95°C, then 13 cycles × (30 seconds at 95°C; 30 seconds at 55 °C; 45 seconds at 72°C), finally 10 minutes at 72°C, 10 °C until halted by user |
| Fungal ITS1 rRNA gene | ITS1F: 5’-CTTGGTCATTTAGAGGAAGTAA-3’; ITS2R: 5’-GCTGCGTTCTTCATCGATGC-3’ | 20 μl reactions consisted of 2 μl 10× Buffer, 2 μl 2.5 mM dNTPs, 0.8μl forward primer(5 μM), 0.8μl reverse primer(5 μM), 0.2 μl rTaq Polymerase, 0.2 μl BSA, 10ng template DNA, ddH_2_O. | 3 minutes at 95°C, then 35 cycles × (30 seconds at 95°C; 30 seconds at 55 °C; 45 seconds at 72°C), finally 10 minutes at 72°C, 10 °C until halted by user |

**Table S2** PERMANOVA, ANOSIM, and MRPP tests showed the significance of bacterial community dissimilarities among different microhabitats, with indices (F, R^2^ or A, and *p*-value) displayed.

| Bacteria | | | | | | | | | |
| --- | --- | --- | --- | --- | --- | --- | --- | --- | --- |
|  | PERMANOVA | | |  | ANOSIM | |  | MRPP | |
| Bray-Curtis | R^2^ | F | *p* |  | R | *p* |  | A | *p* |
| Microhabitat | 0.410 | 12.341 | **0.001** |  | 0.764 | 0.001 |  | 0.215 | **0.001** |
| Site | 0.066 | 2.642 | **0.001** |  | 0.013 | 0.299 |  | 0.012 | 0.074 |
| Microhabitat× Site | 0.192 | 1.930 | **0.001** |  |  |  |  |  |  |
|  | PERMANOVA | | |  | ANOSIM | |  | MRPP | |
| Jaccard | R^2^ | F | *p* |  | R | *p* |  | A | *p* |
| Microhabitat | 0.293 | 6.826 | **0.001** |  | 0.764 | 0.001 |  | 0.135 | **0.001** |
| Site | 0.064 | 1.986 | **0.001** |  | 0.013 | 0.268 |  | 0.009 | **0.044** |
| Microhabitat × Site | 0.214 | 1.658 | **0.001** |  |  |  |  |  |  |

**Table S3** PERMANOVA, ANOSIM, and MRPP tests showed the significance of fungal community dissimilarities among different microhabitats, with indices (F, R^2^ or A, and *p*-value) displayed.

| Fungi | | | | | | | | | |
| --- | --- | --- | --- | --- | --- | --- | --- | --- | --- |
|  | PERMANOVA | | |  | ANOSIM | |  | MRPP | |
| Bray-Curtis | R^2^ | F | *p* |  | R | *p* |  | A | *p* |
| Microhabitat | 0.304 | 8.690 | **0.001** |  | 0.715 | 0.001 |  | 0.146 | **0.001** |
| Site | 0.115 | 4.405 | **0.001** |  | 0.162 | 0.001 |  | 0.043 | **0.001** |
| Microhabitat × Site | 0.231 | 2.205 | **0.001** |  |  |  |  |  |  |
|  | PERMANOVA | | |  | ANOSIM | |  | MRPP | |
| Jaccard | R^2^ | F | *p* |  | R | *p* |  | A | *p* |
| Microhabitat | 0.216 | 4.885 | **0.001** |  | 0.715 | 0.001 |  | 0.088 | **0.001** |
| Site | 0.096 | 2.894 | **0.001** |  | 0.162 | 0.001 |  | 0.028 | **0.001** |
| Microhabitat × Site | 0.245 | 1.841 | **0.001** |  |  |  |  |  |  |

**Table S4** Taxonomic composition of bacterial communities under different microhabitats at the class level. Means with the same letters are not statistically different based on Nemenyi test or Tukey’s HSD as post hoc analysis (*p* < 0.05).

| **Microhabitat-Class** | Bulk soil | Rhizo | Phyllo | Shoots | Roots |
| --- | --- | --- | --- | --- | --- |
| Gammaproteobacteria | 0.2690^c^ | 0.4016^bc^ | 0.5973^ab^ | 0.8178^a^ | 0.8752^a^ |
| Alphaproteobacteria | 0.3857^a^ | 0.2586^ab^ | 0.1942^bc^ | 0.0217^d^ | 0.0665^cd^ |
| Actinomycetes | 0.1398^a^ | 0.1978^a^ | 0.1461^a^ | 0.0184^b^ | 0.0390^b^ |
| Bacilli | 0.0059^ab^ | 0.0243^a^ | 0.0046^ab^ | 0.0875^a^ | 0.0041^b^ |
| Bacteroidia | 0.0115^b^ | 0.0347^a^ | 0.0294^ab^ | 0.0297^ab^ | 0.0144^b^ |
| Thermoleophilia | 0.0708^a^ | 0.0447^ab^ | 0.0138^bc^ | 0.0005^cd^ | 0.0002^d^ |
| Others | 0.0750^a^ | 0.0219^ab^ | 0.0112^bc^ | 0.0009^cd^ | 0.0001^d^ |
| Clostridia | 0^b^ | 0^b^ | 0^b^ | 0.0174^a^ | 0.0002^b^ |
| Acidimicrobiia | 0.0229^a^ | 0.0118^ab^ | 0.0025^bc^ | 0.0009^c^ | 0.0002^c^ |
| Nitrospiria | 0.0060^a^ | 0.0007^ab^ | 0.0001^b^ | 0.0050^ab^ | 0.0001^b^ |
| Acidobacteriae | 0.0134^a^ | 0.0039^ab^ | 0.0007^bc^ | 0.0002^c^ | 0^c^ |

**Table S5** Taxonomic composition of bacterial communities under different microhabitats at the phylum level. Means with the same letters are not statistically different based on Nemenyi test or Tukey’s HSD as post hoc analysis (*p* < 0.05).

| **Microhabitat-Phylum** | Bulk soil | Rhizo | Phyllo | Shoots | Roots |
| --- | --- | --- | --- | --- | --- |
| Pseudomonadota | 0.6548^c^ | 0.6602^c^ | 0.7915^bc^ | 0.8395^ab^ | 0.9417^a^ |
| Actinomycetota | 0.2571^a^ | 0.2628^a^ | 0.1649^a^ | 0.0198^b^ | 0.0394^b^ |
| Bacillota | 0.0059^ab^ | 0.0243^a^ | 0.0046^ab^ | 0.1049^a^ | 0.0042^b^ |
| Bacteroidota | 0.0134^b^ | 0.0356^a^ | 0.0294^ab^ | 0.0303^ab^ | 0.0145^b^ |
| Acidobacteriota | 0.0206^a^ | 0.0044^ab^ | 0.0008^bc^ | 0.0003^c^ | 0^c^ |
| Others | 0.0142^a^ | 0.0029^ab^ | 0.0030^bc^ | 0^c^ | 0^c^ |
| Nitrospirota | 0.0060^a^ | 0.0007^ab^ | 0.0001^b^ | 0.0050^ab^ | 0.0001^b^ |
| Chloroflexota | 0.0156^a^ | 0.0030^ab^ | 0.0002^bc^ | 0^bc^ | 0^c^ |
| Myxococcota | 0.0043^a^ | 0.0023^a^ | 0.0012^ab^ | 0^c^ | 0^bc^ |
| Gemmatimonadota | 0.0040^a^ | 0.0017^a^ | 0.0004^ab^ | 0^b^ | 0^b^ |
| Deinococcota | 0^b^ | 0.0001^b^ | 0.0024^a^ | 0.0002^b^ | 0^b^ |
| Verrucomicrobiota | 0.0032^a^ | 0.0001^ab^ | 0^b^ | 0^b^ | 0^b^ |
| Dependentiae | 0^a^ | 0.0017^a^ | 0^a^ | 0^a^ | 0^a^ |
| Abditibacteriota | 0^b^ | 0^b^ | 0.0014^a^ | 0^b^ | 0^b^ |
| Desulfobacterota | 0.0009^a^ | 0.0002^a^ | 0^a^ | 0^a^ | 0^a^ |

**Table S6** Taxonomic composition of fungal communities under different microhabitats at the class level. Means with the same letters are not statistically different based on Nemenyi test or Tukey’s HSD as post hoc analysis (*p* < 0.05).

| **Microhabitat-Class** | Bulk soil | Rhizo | Phyllo | Shoots | Roots |
| --- | --- | --- | --- | --- | --- |
| Dothideomycetes | 0.1161^b^ | 0.2112^ab^ | 0.3734^a^ | 0.3811^ab^ | 0.2175^ab^ |
| Others | 0.1493^a^ | 0.2132^a^ | 0.1611^a^ | 0.3741^a^ | 0.2495^a^ |
| Leotiomycetes | 0.2856^a^ | 0.1917^ab^ | 0.0502^c^ | 0.1508^bc^ | 0.3814^a^ |
| Sordariomycetes | 0.1275^ab^ | 0.1661^a^ | 0.0522^bc^ | 0.0523^c^ | 0.0501^c^ |
| Eurotiomycetes | 0.0679^a^ | 0.0687^a^ | 0.2958^a^ | 0.0072^b^ | 0.0044^b^ |
| Agaricomycetes | 0.0746^a^ | 0.0414^ab^ | 0.0090^bc^ | 0^c^ | 0.0886^ab^ |
| Mortierellomycetes | 0.1295^a^ | 0.0806^ab^ | 0.0014^bc^ | 0^c^ | 0.0001^c^ |
| Tremellomycetes | 0.0269^a^ | 0.0252^a^ | 0.0184^ab^ | 0.0197^bc^ | 0.0007^c^ |
| Taphrinomycetes | 0.0003^b^ | 0.0009^b^ | 0.0379^a^ | 0.0001^b^ | 0^b^ |
| Orbiliomycetes | 0.0223^a^ | 0.0005^b^ | 0.0002^b^ | 0^b^ | 0.0077^b^ |
| Saccharomycetes | 0^b^ | 0.0002^ab^ | 0.0003^ab^ | 0.0146^a^ | 0^b^ |

**Table S7** Taxonomic composition of fungal communities under different microhabitats at the phylum level. Means with the same letters are not statistically different based on Nemenyi test or Tukey’s HSD as post hoc analysis (*p* < 0.05).

| **Microhabitat-Phylum** | Bulk soil | Rhizo | Phyllo | Shoots | Roots |
| --- | --- | --- | --- | --- | --- |
| Ascomycota | 0.6633^b^ | 0.7325^b^ | 0.8824^a^ | 0.6072^b^ | 0.7000^ab^ |
| Others | 0.0543^b^ | 0.0765^ab^ | 0.0585^b^ | 0.3667^a^ | 0.0830^ab^ |
| Basidiomycota | 0.1186^a^ | 0.0814^a^ | 0.0557^a^ | 0.0197^b^ | 0.2006^a^ |
| Mortierellomycota | 0.1295^a^ | 0.0806^ab^ | 0.0014^bc^ | 0^c^ | 0.0001^c^ |
| Rozellomycota | 0.0229^a^ | 0.0139^a^ | 0.0003^b^ | 0.0010^b^ | 0^b^ |
| Chytridiomycota | 0.0086^a^ | 0.0130^ab^ | 0.0007^abc^ | 0^c^ | 0.0024^bc^ |
| Glomeromycota | 0.0012^a^ | 0.0001^ab^ | 0^ab^ | 0^b^ | 0.0139^a^ |
| Mucoromycota | 0.0008^a^ | 0.0017^a^ | 0.0001^a^ | 0.0010^a^ | 0^a^ |
| Neocallimastigomycota | 0^a^ | 0^a^ | 0^a^ | 0.0027^a^ | 0^a^ |
| Olpidiomycota | 0^a^ | 0.0001^a^ | 0.0007^b^ | 0.0017^a^ | 0^a^ |
| Basidiobolomycota | 0.0009^a^ | 0.0001^a^ | 0.0001^a^ | 0^a^ | 0^a^ |

**Table S8** Major topological properties of co-occurrence networks of each microhabitat.

| **Microhabitat** | **Node** | **Links** | **Average degree** | **Diameter** | **Density** | **Modularity** | **Average Path length** |
| --- | --- | --- | --- | --- | --- | --- | --- |
| Bulk soil | 388 | 4051 | 20.881 | 8 | 0.054 | 0.594 | 3.035 |
| Rhizo | 332 | 1912 | 11.518 | 10 | 0.035 | 0.582 | 4.114 |
| Phyllo | 323 | 2354 | 14.576 | 9 | 0.045 | 0.693 | 3.342 |
| Roots | 51 | 133 | 5.216 | 5 | 0.104 | 0.328 | 1.962 |
| Shoots | 30 | 27 | 1.8 | 4 | 0.062 | 0.748 | 1.967 |
